# Neuropsychiatric comorbidities in Huntington’s and Parkinson’s Disease: A United States claims database analysis

**DOI:** 10.1002/acn3.51252

**Published:** 2020-11-20

**Authors:** Lianna Ishihara, David Oliveri, Edward J. Wild

**Affiliations:** ^1^ Roche Products Ltd Welwyn Garden City UK; ^2^ Genesis Research Hoboken NJ USA; ^3^ Huntington’s Disease Centre UCL Queen Square Institute of Neurology University College London London UK

## Abstract

**Objective:**

Huntington’s disease is a rare, genetic, neurodegenerative disease characterized by a triad of cognitive, behavioral, and motor symptoms. The condition gradually results in increasing disability, loss of independence, and ultimately death. Our objective was to use United States claims data (which offer valuable insight into the natural history of disease) to compare the prevalent comorbidities of people with Huntington’s disease against matched controls with Parkinson’s disease or with no major neurodegenerative diseases (general population controls). We also assess medication use in people with Huntington’s disease.

**Methods:**

This was a retrospective, observational study using data from the IBM MarketScan^®^ Databases. Cases and controls were matched 1:1, and comorbidities were analyzed in each group during 2017. Medications were also assessed in the Huntington’s disease cohort. Eligible cases had ≥ 2 diagnostic codes for Huntington’s disease; controls had ≥ 2 codes for Parkinson’s disease (with no record of Huntington’s disease), or, for general population controls, no record of Huntington’s disease, Parkinson’s disease, amyotrophic lateral sclerosis, or dementia.

**Results:**

A total of 587 matched individuals were assessed in each cohort. Depression and anxiety were more common in Huntington’s disease versus Parkinson’s disease (odds ratios: 1.51 and 1.16, respectively). Other conditions more common in Huntington’s disease included dementia, communication/speech problems, dysphagia, and falls. The use of antidepressant (59.9%) and antipsychotic (39.5%) medications was frequent among Huntington’s disease cases.

**Interpretation:**

These data highlight the prevalence of psychiatric, cognitive, communication, swallowing, and mobility problems in people with Huntington’s disease, underscoring the need for holistic expert care of these individuals.

## Introduction

Huntington’s disease (HD) is a rare, autosomal‐dominant neurodegenerative disease that manifests as a triad of cognitive, behavioral, and motor symptoms. HD results in increasing disability, loss of independence, and death, with a median survival of 15 years after the onset of motor symptoms.^1^ The disease is caused by a cytosine‐adenine‐guanine (CAG) trinucleotide repeat expansion in the *HTT* gene, resulting in the production of a toxic mutant huntingtin (mHTT) protein.^2^, ^3^, ^4^ The neuropathological abnormalities associated with HD appear to be caused by a dominant gain‐of‐function in mHTT.^5^ Reducing the synthesis of this protein targets the primary disease mechanism; however, there are currently no treatments that can cure or modify HD progression.

The prevalence of manifest (symptomatic) HD is estimated to be ~ 10 in 100,000,^6^, ^7^, ^8^, ^9^ although it is likely that prevalence rates are underestimated due to the unavailability of genetic testing or social stigma.^8^ There is evidence that the prevalence in Western countries has increased over the past 50 years,^10^ likely due to increases in life expectancy and the advent of diagnostic testing.^10^, ^11^ The number of individuals at risk of inheriting the disease (i.e., first‐degree relatives of people with HD with unknown genetic status) is estimated to fall between 30 and 47 per 100,000 in the Western population.^7^, ^9^

Cognitive disturbances in HD are characterized by a reduction in the speed and flexibility of mental processing, as well as decreased attention, visuospatial function, and emotional recognition.^5^ These changes can occur years before the diagnosis of motor onset, and deteriorate steadily as the disease progresses.^12^ Behavioral and neuropsychiatric changes are less predictable. Common psychiatric conditions among people with HD are irritability, depression, and anxiety.^13^, ^14^ The occurrences of these conditions do not relate to any specific stage of the disease,^15^ and may occur many years before a clinical diagnosis of HD is made.^16^ The motor symptoms of HD include both impairment of voluntary movements and the emergence of involuntary movements (chorea), which result in reduced manual dexterity, slurred speech, swallowing difficulties, problems with balance, and falls. Motor symptoms in HD are initially subtle and progress until virtually all movement‐associated functions are affected.^5^, ^17^ Although the clinical diagnosis of HD relies on the manifestation of motor symptoms,^5^, ^18^ cognitive and psychiatric changes place the greatest burden on families, are most associated with functional decline and can be predictive of institutionalization.^12^ Almost all people with HD will manifest disease‐specific personality and behavioral changes, as part of what might be termed a hypofrontal or dysexecutive syndrome. These changes are characterized by apathy, irritability, impulsivity, and obsessiveness, with potentially severe consequences in terms of marital, social, and economic well‐being.^19^ Suicide in HD has been reported to be as high as 13%, a 7‐ to 12‐fold increase above that in the general population.^20^

Parkinson’s disease (PD) is a progressive, highly debilitating neurodegenerative disorder which has a significant impact on quality of life and increases the burden on caregivers and families.^21^ The mean age of onset is 60–65 years.^22^, ^23^ Although the clinical diagnosis of PD is currently based only on motor symptoms, nonmotor symptoms can often precede diagnosis by several years.^24^, ^25^ Increasingly, nonmotor symptoms among people with PD is an area of focus in the literature,^26^, ^27^, ^28^, ^29^ but this does not receive as much attention in HD.

Insurance claims data from the United States offer a valuable insight into the natural history of HD. IBM MarketScan^®^ has previously been used to explore differences in healthcare interventions and direct medical costs among people with HD by disease stage.^30^, ^31^ The aim of this retrospective, matched cohort study was to compare the prevalent comorbidities of people with HD, which may be true features of the disease or coexisting independent conditions, against matched controls – people with PD or with no major neurodegenerative diseases (PD, amyotrophic lateral sclerosis [ALS], or dementia) – using US claims data. Patterns of medication use were also analyzed in the HD cohort. It is hoped that this will provide insights into the natural history of HD and the real‐world management of this condition.

## Methods

### Study design

This was a retrospective, observational, matched cohort study using secondary data from the IBM MarketScan^®^ Commercial and Multi‐State Medicaid Databases.

To limit the risk of selection bias, the study population comprised all eligible HD cases, which were then matched to controls with PD or with no neurodegenerative diseases (general population [GP] controls; without HD, PD, ALS, or dementia). Cases and controls were matched 1:1 by data source (Commercial or Medicaid), gender, birth year, and months of enrollment at baseline (grouped into 3‐month windows). Comorbidities were assessed in HD cases and matched controls, whereas medication use was analyzed only in the HD cohort.

The most recently available calendar year of data was used to define the analysis period (Jan 1, 2017–Dec 31, 2017). The first day of the analysis period was considered the index date for cases and controls.

### Study population

All cases and controls were identified in the IBM MarketScan^®^ Commercial and Multi‐State Medicaid Databases. Eligible individuals were aged between 2 and 64 years on the index date, with full coverage for medical care in 2017 (HD cases were also required to have full medication coverage for the whole study period, plus 2 months prior to the index date).

Eligible HD cases had ≥ 2 International Classification of Diseases (ICD)‐9/10 diagnostic codes for HD (333.4/G10, respectively), with ≥ 1 code on or before the index date. Eligible PD controls had ≥ 2 diagnostic codes for PD on or before the index date (individuals with a record of a claim for HD were excluded from this group). GP controls were those without any record of a claim for HD, PD, ALS or dementia; a random 1% sample of this cohort was used in the study. Across all cohorts, individuals with < 6 months’ continuous enrollment during the 6 months prior to the index date were excluded.

Subanalyses were performed in individuals aged 2–17, 18–24, 25–49, and ≥ 50 years on the index date.

### Study analyses

The primary analysis compared the relative prevalence of comorbidities (which may be true features of the disease or coexisting independent conditions) in HD cases and matched controls (either with PD, or without any major neurodegenerative disease), based on records in the IBM MarketScan^®^ Databases. A case or control was considered to have a comorbidity if they had ≥ 1 record of this condition within the time period being considered. The prevalence of comorbidities was reported for two periods: (1) the analysis period, and (2) the analysis period plus any time before (within the period of continuous enrollment). All comorbidities available in the ICD‐9/10 categorization were reported by subheading. In addition, several comorbidities/symptoms of interest were specifically defined using ICD‐9/10 codes and reviewed by a clinician (Table S1).

Since the study cohorts were defined differently for the primary analysis (i.e., full medication coverage was required for HD cases but not for PD/GP controls), a sensitivity analysis was conducted in which comorbidities were assessed without the requirement for full medication coverage in HD cases. In the interests of brevity, the results of this analysis are reported only for the analysis period.

Medication use was analyzed for HD cases during the analysis period. An individual was considered to be using a medication if they were issued a prescription at any time within this period. Medications were reported by drug class.

### Statistical analysis

Demographic characteristics are summarized as the frequency of individuals in each group (n, %) for categorical data, and as mean with standard deviation (SD), or median with upper/lower quartiles (Q1–Q3) or range (minimum–maximum), for continuous data.

To assess the relative prevalence of comorbidities in HD cases and matched controls, unstratified matched analyses were conducted via conditional logistic regression, and stratified analyses via the Fisher Exact tests. The outputs of these analyses were odds ratios (ORs) with 95% confidence intervals (CIs) for the period of interest. ORs and 95% CIs are reported without p‐values. Since these are exploratory analyses, and sample sizes were not based on statistical considerations, there was no adjustment for multiple testing. ORs are presented only where there were ≥ 5 individuals in both the case and control populations with a record of the comorbidity during the time period of interest. The results are presented for the 10 highest ORs comparing cases and controls.

The prevalence of medication use in HD cases was calculated as the number of eligible individuals with a record of use during the analysis period, divided by the total number of eligible individuals. This is reported as the frequency of people (n, %) with a claim for each drug class during the analysis period.

SAS (version 9.4) and Teradata SQL were used to extract the cohorts, derive analysis variables, and analyze the data. Given the nature of this study (secondary use of routinely collected healthcare data), no provisions were made for missing data.

## Results

### Disposition and demographics

**Figure 1:**
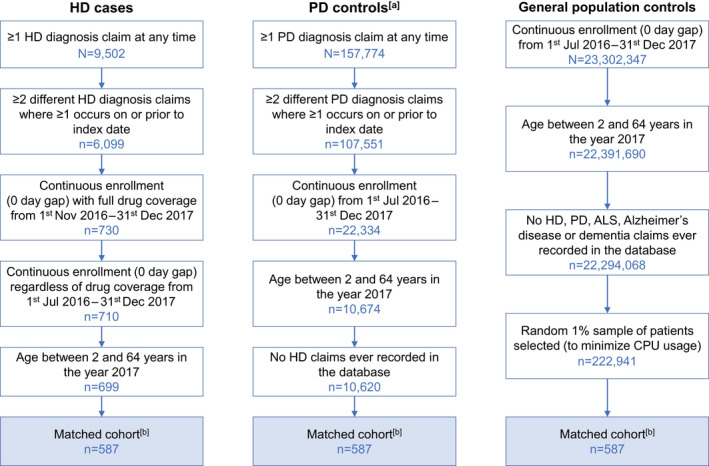
Study population disposition. (a) Excluding the 332.1 and G21.x diagnostic codes; (b) Cases and controls were matched 1:1 by data source (Commercial or Medicaid), gender, birth year, and months of enrollment at baseline (grouped into 3‐month windows).

The HD cohort comprised 699 cases in total, whereas the PD and GP cohorts comprised 10,620 and 222,941 individuals, respectively (Figure 1). Eligible people from each cohort were matched 1:1, yielding a total of 587 matched patients in each group.

In the unmatched cohorts, there was a greater proportion of females among HD cases compared to PD controls (59.5% vs. 44.0%). HD cases were also younger on average than PD controls (median age: 50 vs. 59 years) (Table S2). While data for HD cases were sourced about equally from the commercial and Medicaid databases (50.9% and 49.1%, respectively), for PD controls, data were more frequently sourced from the commercial database (58.0%, vs. 42.0% from Medicaid) (Table S2).

In the matched cohorts, most individuals (59.5%) were aged ≥ 50 years (median age: 52), and the majority were female (59.3%). Data from matched individuals were sourced about equally from the commercial and Medicaid databases (53.5% and 46.5%, respectively) (Table S2). Crucially, the matched HD cohort was demographically similar to, and thus representative of, the unmatched population (Table S2).

### Comorbidities

#### Prespecified comorbidities

**Figure 2:**
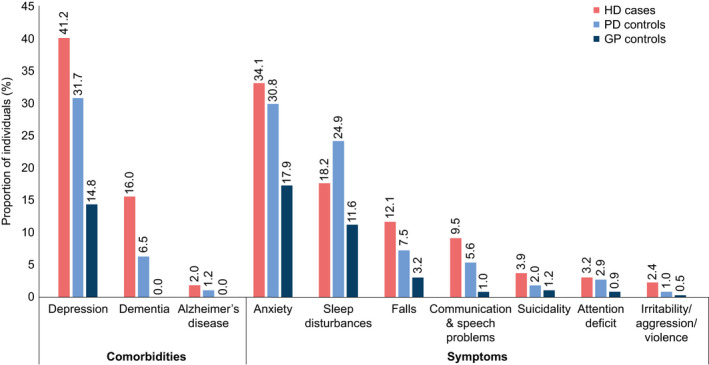
Frequency of prespecified comorbidities/symptoms during the analysis period. *N* = 587 for each cohort. Data are presented for the 10 most frequent comorbidities among HD cases.

Of the prespecified comorbidities, depression and anxiety were the most frequently reported during the analysis period, and were more common in the HD and PD cohorts compared with the general population (Figure 2).

**Figure 3:**
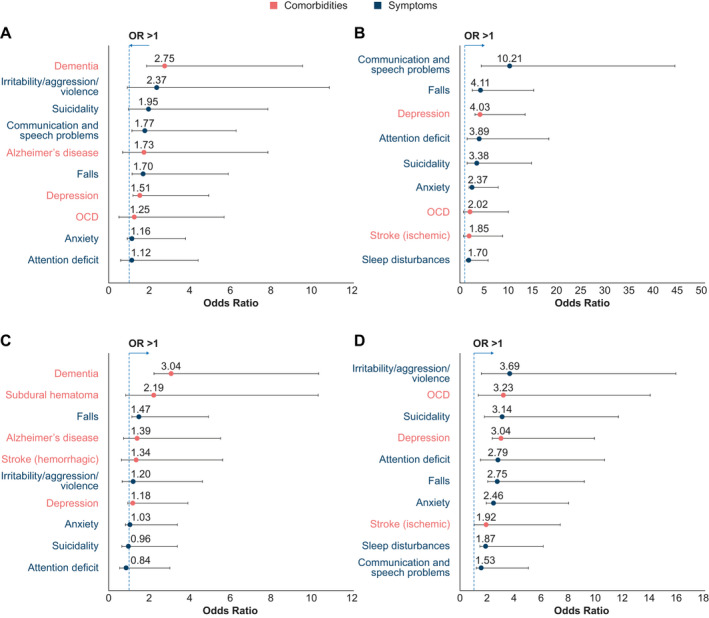
Prespecified comorbidities/symptoms in HD cases versus controls (odds ratios) (A) HD cases versus PD controls (analysis period only) (B) HD cases versus GP controls (analysis period only) (C) HD cases versus PD controls (analysis period and prior) (D) HD cases versus GP controls (analysis period and prior). *N* = 587 for each cohort. In each graph, the ten greatest odds ratios are presented, with 95% confidence intervals, from the panel of prespecified comorbidities that were reported in ≥ 5 cases and ≥ 5 controls. Odds ratios lower than those shown in the figure are presented in Table S11. Some prespecified comorbidities, while frequent among HD cases, were not sufficiently frequent in the respective control population to allow calculation of an odds ratio. These were, for Panel B: dementia, irritability/aggression/violence, and Alzheimer’s disease, and for Panel D: dementia. GP: general population; HD: Huntington’s disease; OCD: obsessive–compulsive disorder; PD: Parkinson’s disease.

Dementia was more likely to occur in HD cases relative to PD controls (OR [95% CI]: 2.75 [1.85–4.09]) (Figure 3A), and depression (1.51 [1.19–1.92]) and anxiety (1.16 [0.91–1.48]) were also more common among HD cases (Figure 3A). Stratification by age group revealed a number of comorbidities that were more likely to occur in HD cases than PD controls, particularly among those aged 25–49 or ≥ 50 years (Table S3). In both groups, however, dementia was associated with the highest ORs.

Compared to the GP cohort, communication and speech problems were more likely to occur in HD cases (10.21 [4.36–23.90]) (Figure 3B). This was also true when considering the subgroup of individuals aged ≥ 50 years; however, in those aged 25–49 years, depression was associated with the greatest OR (Table S4).

When considering both the analysis period and any time prior (within the period of continuous enrollment), depression and anxiety remained the most frequently reported comorbidities, particularly in the HD and PD cohorts. Dementia remained more likely to occur in HD cases relative to PD controls (3.04 [2.21–4.16]) (Figure 3C), and this was also true in the subgroups aged 25–49 and ≥ 50 years (Table S5). Depression (1.18 [0.93–1.49]) and anxiety (1.03 [0.82–1.29]) also remained more common in the HD (vs. PD) cohort (Figure 3C). Irritability/aggression/violence were reported more frequently in HD cases versus GP controls (3.69 [1.58–8.59]) (Figure 3D); however, depression and suicidality were associated with highest ORs upon stratification by age group (Table S6).

#### Overall comorbidities

**Figure 4:**
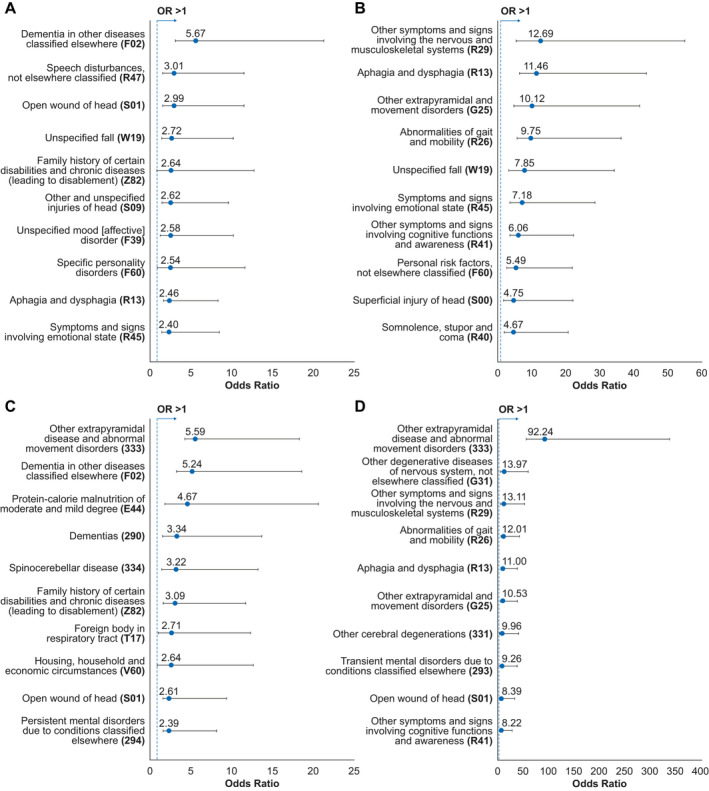
Overall comorbidities in HD cases versus controls (odds ratios) (A) HD cases versus PD controls (analysis period only) (B) HD cases versus GP controls (analysis period only) (C) HD cases versus PD controls (analysis period and prior) (D) HD cases versus GP controls (analysis period and prior). *N* = 587 for each cohort. In each graph, the ten greatest odds ratios are presented, with 95% confidence intervals, from the overall panel of comorbidities that were reported in ≥ 5 cases and ≥ 5 controls. Odds ratios lower than those shown in the figure are presented in Table S12. GP: general population; HD: Huntington’s disease; PD: Parkinson’s disease.

When considering the broader panel of comorbidities, dementia remained more prevalent in HD cases versus PD controls during the analysis period (5.67 [3.22–9.99]) (Figure 4A), whereas nervous and musculoskeletal symptoms were more common relative to GP controls (12.69 [5.46–29.48]) (Figure 4B). Dementia was not captured in the latter group, since this was an exclusion criterion for GP controls. Stratification by age group revealed some heterogeneity: compared to PD controls, HD cases aged 25–49 were more likely to have a record of unspecified head injuries, whereas dementia was more common in those aged ≥ 50 years (Table S7). Compared to GP controls, aphagia and dysphagia were more likely to be reported in HD cases aged 25–49 or ≥ 50 years (Table S8).

When considering both the analysis period and any time prior, extrapyramidal disease and abnormal movement disorders were associated with the highest ORs in HD cases compared to both PD and GP controls (5.59 [4.33–7.20] and 92.24 [56.79–149.82], respectively) (Figure 4C–D). Multiple categories of dementia were more strongly associated with HD compared with PD (Figures 4A, C), corroborating what was observed with the prespecified list.

Extrapyramidal disease and abnormal movement disorders were the most frequently occurring comorbidity in HD cases versus PD controls within the 18–24 subgroup, whereas dementia was associated with the greatest ORs in the 25–49 and ≥ 50 subgroups (Table S9). In the HD versus GP comparison, depressive disorder had the highest OR among individuals aged 18–24 years, and extrapyramidal disease/abnormal movement disorders in those aged 25–49 or ≥ 50 years (Table S10).

#### Sensitivity analysis

The results of the sensitivity analysis – in which the requirement for full medication coverage was removed across all cohorts – largely reflected those of the primary analysis. Of the prespecified comorbidities, depression and anxiety were the most frequently reported during the analysis period, and were more common in people with HD (45.8% and 37.2%, respectively) or PD (38.0% and 35.1%) versus GP controls (18.5% and 17.7%). Compared with PD controls, dementia was more common in the HD cohort during the analysis period (2.83 [2.18–3.68]). Depression (1.38 [1.15–1.65]) and anxiety (1.09 [0.91–1.31]) were also more common in HD versus PD, though the magnitude of these differences were reduced compared with the primary analysis. In the broader panel of comorbidities, dementia remained more common in HD versus PD during the analysis period (4.59 [3.23–6.52]) and lack of coordination was more common versus GP controls (23.96 [10.51–54.62]).

### Medication Use

**Figure 5:**
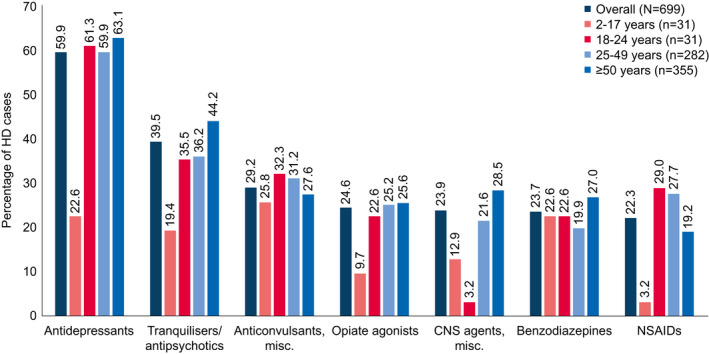
Medication use among HD cases (analysis period only). Unmatched HD cohort. Includes medications reported in ≥ 20% of HD cases. HD: Huntington’s disease; NSAID: nonsteroidal anti‐inflammatory drug

Antidepressants and antipsychotics were the most frequently used drug classes within the HD cohort (59.9% and 39.5%, respectively), and the same pattern was evident upon stratification by age group (Figure 5).

## Discussion

The aim of this retrospective, matched cohort study was to compare the prevalent comorbidities of people with HD against matched PD or GP controls, and to assess patterns of medication use in HD cases, with a view to gathering insights into the natural history of HD and standard practices used in a real‐world setting.

Examination of US claims data revealed that dementia, psychiatric comorbidities, communication and speech problems, dysphagia, and falls are common in people with HD relative to both the general population and PD. Many of these conditions are expected consequences of the cognitive and motor impairment associated with HD. Similarly, PD is associated with nonmotor symptoms – including psychiatric disturbances – which can often precede diagnosis by several years,^24^, ^25^ and are well‐documented in the literature.^26^, ^27^, ^28^, ^29^ In our study, dementia, in particular, was consistently strongly associated with HD, regardless of the time period or age group under consideration; this was supported in a sensitivity analysis that excluded the requirement for full medication coverage in the HD cohort. These data support the outcomes of a recent comparative study of people with HD versus PD,^32^ which revealed a panel of nonmotor symptoms that were more common in the former group, including attentional deficits, dysphagia, memory complaints, depression, and falls.

Extrapyramidal disease and abnormal movement disorders were associated with the highest ORs in HD cases compared with both PD and GP controls, when considering both the analysis period and any time prior. This could suggest that people with HD more frequently receive a generalized diagnosis for a period of time before the diagnosis of HD is confirmed (e.g., with genetic testing), whereas patients with PD tend to present with a set of symptoms that quickly lead to a definitive diagnosis.

The use of antidepressants and antipsychotics was also common among HD cases, indicating a relatively high psychiatric burden in this population. These findings are aligned with previous reports, although older studies have limitations such as small datasets or larger self‐selected cohorts.^33^, ^34^ Psychiatric disorders are known to be an integral component of HD progression.^13^, ^14^ However, the perception of depressive symptoms as a “natural consequence” of having the disease may lead to underdiagnosis or inadequate treatment of this condition in people with HD.^16^, ^35^ This issue extends to other psychiatric disturbances – such as mania, obsessive–compulsive disorder, and schizophrenia – all of which have parallels with HD in their symptomatic presentation,^16^ and thus may not be identified and treated as separate conditions. It is hoped that the data presented in this report will shed light on the widespread presence of such comorbidities among people with HD. Nevertheless, it is important to note that some instances of antipsychotic drug use in this population may have related to control of motoric symptoms, such as chorea.^36^

Our analyses of the frequency of the prespecified comorbidities/symptoms showed only a threefold increase in suicidality in HD cases compared with the GP controls, which are lower than rates of suicidal ideation reported in the literature.^20^ There could be a number of reasons for this discrepancy. Inaccurate reporting is a possibility; suicidal ideation is generally under reported in the general population as a whole and coding errors are common for suicide and suicide attempts. Some studies suggest that suicidal ideation decreases immediately after the diagnosis of HD, whereas uncertainty around diagnosis can potentially increase ideation.^37^ As the HD cohort in this study had ≥ 2 diagnostic codes for HD there is a possibility that this group had reduced levels of suicidal ideation. A recent systematic review highlights that inconsistencies in the terminology for suicide and suicidal ideation and variable assessment tools mean that it is still challenging to compare different studies on this topic in HD and more research is needed.^38^ Hubers AA et al. found that 8% of HD mutation carriers endorsed suicidal ideation, which is more aligned with our findings; however, they also acknowledge that their study had limitations resulting in probable underestimation.^39^

While data for HD cases were sourced about equally from the commercial and Medicaid databases, for PD controls, data were more frequently sourced from the commercially insured population. The reasons for this discrepancy remain unclear, though it has been shown that the commercial database captures an even distribution across HD disease stages, whereas most people with HD in the Medicaid population have late‐stage disease.^30^

The IBM MarketScan® Commercial Database contains data for several million individuals annually, encompassing employees, their spouses, and dependents who are covered by employer‐sponsored private health insurance in the United States. The IBM MarketScan® Multi‐State Medicaid Database contains the medical, surgical, and prescription drug experience of more than 40 million enrollees from multiple states. The strengths of these databases lie in their large sample sizes and strong longitudinal tracking at the individual level, as long as the person remains enrolled with the participating plans. In addition, the gender, age, and geographic distributions of the database population are representative of Americans covered by health insurance, and the database captures the continuum of care in multiple settings, including physician office visits, hospital stays, and pharmacies.

A potential limitation of the reliance on secondary data is the scope for inaccurate reporting of diagnoses or medication usage. To mitigate the possibility of disease misclassification due to reporting or coding errors, two diagnostic codes were required to identify the HD and PD cohorts in this study. The comorbidities of interest (Table S1) were prespecified to investigate whether such diagnoses may be recorded as possible symptoms or early manifestations of disease; however, our data cannot distinguish between misdiagnoses and true additional or secondary diagnoses. It should also be noted that comorbidities were only analyzed when there was a record of the diagnosis in question for ≥ 5 cases and ≥ 5 controls. Since some conditions warranted exclusion from the GP cohort (e.g., dementia), this may have precluded the analysis of comorbidities that were highly prevalent in the HD cohort. The use of this threshold also led to some apparent “discrepancies” between the most prevalent comorbidities in the overall population versus age‐specific subpopulations (since the cut‐off of ≥ 5 cases and ≥ 5 controls is harder to meet in smaller groups of patients). Thus, any such “discrepancies” were likely an artifact of these selection criteria.

A further limitation lies in the comparison between HD and PD itself. We include this comparison with PD, a more prevalent disease, to contextualize our results. While both HD and PD are characterized by motor and nonmotor symptoms, the typical age of onset is lower in the HD population, and age of onset can influence disease course.^23^, ^40^ Cases and controls were matched by age at index, rather than onset, and people with PD aged ≥ 65 years were excluded due to low prevalence of HD in this age bracket. Additionally, a number of people in the PD cohorts were aged under 25 years at the index date, whereas Parkinson’s disease typically develops in later life. The exclusion of people with PD aged ≥ 65 years is likely to have contributed to a higher proportion of younger people in the PD cohorts, although the actual percentage of people under the age of 25 years at index date with ≥ 2 diagnostic codes for PD was only 1% in the unmatched cohort and 4.6% in the matched cohort. The literature suggests that in 3% to 5% of cases of PD, symptoms start before the age of 40 years, and in Japan early‐onset PD has been reported in 10% to 20% of cases.^22^ It remains unclear to what extent these factors may have confounded the comparison of comorbidities between the two groups.

Collectively, the data presented here provide insight into the natural history and real‐world treatment of HD and comorbid conditions, and point toward a significant burden of psychiatric, cognitive, communication, swallowing, and mobility problems in people with HD. Moreover, since only about 40% of people with symptomatic HD in the United States are under the care of specialists,^41^ the burden may be substantially higher than that presented here. Collectively, these findings underscore the need for holistic expert care of people with HD and highlight the need for future studies investigating the treatment of anxiety, depression, and other comorbid conditions in this population.

## Data Sharing Statement

This was a secondary analysis of routinely collected healthcare data from the IBM MarketScan^®^ Commercial and Multi‐State Medicaid Databases. MarketScan is a registered trademark of Truven Health Analytics Inc., an IBM Company.

## Conflict of Interest

During the conduct of the work and most of the manuscript development, **LI** was employed by F. Hoffmann‐La Roche where she is still a shareholder. She is now employed by Bristol Myers Squibb. **DO** is an employee of Genesis Research, which received consulting fees from Roche. Via UCL Consultants Ltd, a wholly‐owned subsidiary of University College London, **EJW** has served on scientific advisory boards for Novartis, F. Hoffmann–La Roche, Ionis, Shire, Wave Life Sciences, PTC Therapeutics, and Mitoconix. His research team has received academic grant funding from F. Hoffmann‐La Roche. His host healthcare institution, University College London Hospitals NHS Foundation Trust, has received funding from F. Hoffmann‐La Roche for the conduct of clinical trials in Huntington’s disease.

## Authors’ Contributions

LI, DO, and EJW involved in the substantial contributions to study conception and design, analysis and interpretation of the data, drafting the article or revising it critically for important intellectual content, and final approval of the version of the article to be published.

## Supporting information


**Table S1**. Comorbidities/symptoms of interest
**Table S**2. Study population demographics. HD: Huntington’s disease; PD: Parkinson’s disease; SD: standard deviation.
**Table S**3. Prespecified comorbidities stratified by age group: HD cases versus PD controls (analysis period only). Odds ratios are presented with 95% confidence intervals, only for prespecified comorbidities that were reported in ≥ 5 cases and ≥ 5 controls. Only the highest 10 odds ratios are presented for each age group. CI: confidence interval; HD: Huntington’s disease; OCD: obsessive–compulsive disorder; OR: odds ratio; PD: Parkinson’s disease.
**Table S**4. Prespecified comorbidities stratified by age group: HD cases versus GP controls (analysis period only). Odds ratios are presented with 95% confidence intervals, only for prespecified comorbidities that were reported in ≥ 5 cases and ≥ 5 controls. Only the highest 10 odds ratios are presented for each age group. CI: confidence interval; GP: general population; HD: Huntington’s disease; OCD: obsessive–compulsive disorder; OR: odds ratio.
**Table S**5. Prespecified comorbidities stratified by age group: HD cases versus PD controls (analysis period and prior). Odds ratios are presented with 95% confidence intervals, only for prespecified comorbidities that were reported in ≥ 5 cases and ≥ 5 controls. Only the highest 10 odds ratios are presented for each age group. CI: confidence interval; HD: Huntington’s disease; OCD: obsessive–compulsive disorder; OR: odds ratio; PD: Parkinson’s disease.
**Table S**6. Prespecified comorbidities stratified by age group: HD cases versus GP controls (analysis period and prior). Odds ratios are presented with 95% confidence intervals, only for prespecified comorbidities that were reported in ≥ 5 cases and ≥ 5 controls. Only the highest 10 odds ratios are presented for each age group. CI: confidence interval; HD: Huntington’s disease; OCD: obsessive–compulsive disorder; OR: odds ratio.
**Table S**7. Overall comorbidities stratified by age group: HD cases versus PD controls (analysis period only). Odds ratios are presented with 95% confidence intervals, only for comorbidities that were reported in ≥ 5 cases and ≥ 5 controls. Only the highest 10 odds ratios are presented for each age group. CI: confidence interval; HD: Huntington’s disease; ICD: International Classification of Diseases; OCD: obsessive–compulsive disorder; OR: odds ratio; PD: Parkinson’s disease.
**Table S**8. Overall comorbidities stratified by age group: HD cases versus GP controls (analysis period only). Odds ratios are presented with 95% confidence intervals, only for comorbidities that were reported in ≥ 5 cases and ≥ 5 controls. Only the highest ten odds ratios are presented for each age group. CI: confidence interval; GP: general population; HD: Huntington’s disease; ICD: International Classification of Diseases; OCD: obsessive–compulsive disorder; OR: odds ratio.
**Table S**9. Overall comorbidities stratified by age group: HD cases versus PD controls (analysis period and prior). Odds ratios are presented with 95% confidence intervals, only for comorbidities that were reported in ≥ 5 cases and ≥ 5 controls. Only the highest ten odds ratios are presented for each age group. CI: confidence interval; GP: general population; HD: Huntington’s disease; ICD: International Classification of Diseases; OCD: obsessive–compulsive disorder; OR: odds ratio; PD: Parkinson’s disease.
**Table S**10. Overall comorbidities stratified by age group: HD cases versus GP controls (analysis period and prior). Odds ratios are presented with 95% confidence intervals, only for comorbidities that were reported in ≥ 5 cases and ≥ 5 controls. Only the highest ten odds ratios are presented for each age group. CI: confidence interval; GP: general population; HD: Huntington’s disease; ICD: International Classification of Diseases; OCD: obsessive–compulsive disorder; OR: odds ratio.
**Table S**11. Odds ratios for prespecified comorbidities not listed in Figure 3. CI: confidence interval; OR: odds ratio.
**Table S**12. Odds ratios for overall comorbidities not listed in Figure 4. CI: confidence interval; OR: odds ratio.
