# Supplementary material for: Neuropsychiatric comorbidities in Huntington’s and Parkinson’s Disease: A United States claims database analysis
Source: Ann Clin Transl Neurol. 2020 Nov 20;8(1):126–37. doi: 10.1002/acn3.51252 (PMC7818185; doi:10.1002/acn3.51252)
Supplement: Supplementary file 1 — Table S1. Comorbidities/symptoms of interest Table S2. Study population demographics. HD: Huntington’s disease; PD: Parkinson’s disease; SD: standard deviation. Table S3. Prespecified comorbidities stratified by age group: HD cases versus PD controls (analysis period only). Odds ratios are presented with 95% confidence intervals, only for prespecified comorbidities that were reported in ≥ 5 cases and ≥ 5 controls. Only the highest 10 odds ratios are presented for each age group. CI: confidence interval; HD: Huntington’s disease; OCD: obsessive–compulsive disorder; OR: odds ratio; PD: Parkinson’s disease. Table S4. Prespecified comorbidities stratified by age group: HD cases versus GP controls (analysis period only). Odds ratios are presented with 95% confidence intervals, only for prespecified comorbidities that were reported in ≥ 5 cases and ≥ 5 controls. Only the highest 10 odds ratios are presented for each age group. CI: confidence interval; GP: general population; HD: Huntington’s disease; OCD: obsessive–compulsive disorder; OR: odds ratio. Table S5. Prespecified comorbidities stratified by age group: HD cases versus PD controls (analysis period and prior). Odds ratios are presented with 95% confidence intervals, only for prespecified comorbidities that were reported in ≥ 5 cases and ≥ 5 controls. Only the highest 10 odds ratios are presented for each age group. CI: confidence interval; HD: Huntington’s disease; OCD: obsessive–compulsive disorder; OR: odds ratio; PD: Parkinson’s disease. Table S6. Prespecified comorbidities stratified by age group: HD cases versus GP controls (analysis period and prior). Odds ratios are presented with 95% confidence intervals, only for prespecified comorbidities that were reported in ≥ 5 cases and ≥ 5 controls. Only the highest 10 odds ratios are presented for each age group. CI: confidence interval; HD: Huntington’s disease; OCD: obsessive–compulsive disorder; OR: odds ratio. Table S7. Overall comorbidities stratified by age g [file ACN3-8-126-s001.docx]

**SUPPLEMENTARY DATA**

Supplementary Table 1. Comorbidities/symptoms of interest

| Comorbidities   - Alzheimer’s disease - Dementia - Depression - Stroke (hemorrhagic) - Stroke (ischemic) - ALS - OCD - Subdural haematoma - Myocardial infarction   Symptoms   - Anxiety - Apathy - Attention deficit - Communication & speech problems - Falls - Irritability/aggression/violence - Sleep disturbances - Suicidality |
| --- |

Supplementary Table 2. Study population demographics

| ***Characteristic,***  *n (%) unless otherwise specified* | **Cohort** | | | | |
| --- | --- | --- | --- | --- | --- |
|  | **Unmatched HD**  **N=699** | **Matched HD**  **N=587** | **Unmatched PD**  **N=10,620** | **Matched PD**  **N=587** | **Matched gen. pop.**  **N=587** |
| **Gender** | | | | | |
| Male | 283 (40.5) | 239 (40.7) | 5,944 (56.0) | 239 (40.7) | 239 (40.7) |
| Female | 416 (59.5) | 348 (59.3) | 4,676 (44.0) | 348 (59.3) | 348 (59.3) |
| **Index age (years)** | | | | | |
| 2–17 | 31 (4.4) | 10 (1.7) | 41 (0.4) | 10 (1.7) | 10 (1.7) |
| 18–24 | 31 (4.4) | 17 (2.9) | 67 (0.6) | 17 (2.9) | 17 (2.9) |
| 25–49 | 282 (40.3) | 211 (36.0) | 1,285 (12.1) | 211 (36.0) | 211 (36.0) |
| ≥50 | 355 (50.8) | 349 (59.5) | 9,227 (86.9) | 349 (59.5) | 349 (59.5) |
| Mean (SD) | 46.6 (13.5) | 49.6 (11.5) | 56.8 (7.9) | 49.6 (11.5) | 49.6 (11.5) |
| Median (Q1–Q3) | 50 (39–57) | 52 (44–58) | 59 (54–62) | 52 (44–58) | 52 (44–58) |
| Min–Max | 2–64 | 5–64 | 4–64 | 5–64 | 5–64 |
| **Data source** | | | | | |
| MarketScan Commercial Database | 356 (50.9) | 314 (53.5) | 6,157 (58.0) | 314 (53.5) | 314 (53.5) |
| MarketScan Multi-State Medicaid Database | 343 (49.1) | 273 (46.5) | 4,463 (42.0) | 273 (46.5) | 273 (46.5) |
| **Enrollment history (years)** | | | | | |
| Mean (SD) | 4.8 (3.7) | 5.2 (3.8) | 5.3 (4.0) | 7.5 (5.5) | 8.0 (5.9) |
| Median (Q1–Q3) | 4 (2.3–5.0) | 4 (3.0–5.8) | 4 (2.5–6.0) | 5.0 (4.0–12.0) | 5.0 (4.0–16.0) |
| Min–Max | 0.5–17.0 | 0.5–17.0 | 0.5–17.0 | 0.6–17.0 | 0.5–17.0 |

HD: Huntington’s disease; PD: Parkinson’s disease; SD: standard deviation.

Supplementary Table 3. Prespecified comorbidities stratified by age group: HD cases versus PD controls (analysis period only)

| **Comorbidity** | **OR (95% CI)** |
| --- | --- |
| **18–24 years (n=34)** | |
| Depression | 1.68 (0.41–6.96) |
| **25–49 years** **(n=422)** | |
| Dementia | 5.01 (2.02–12.41) |
| Falls | 2.58 (1.20–5.54) |
| Suicidality | 2.27 (0.77–6.64) |
| Depression | 1.86 (1.25–2.79) |
| Communication & speech problems | 1.59 (0.77–3.30) |
| Anxiety | 1.51 (1.00–2.28) |
| Attention deficit | 1.35 (0.46–3.95) |
| Sleep disturbances | 0.83 (0.51–1.35) |
| OCD | 0.83 (0.25–2.76) |
| **≥50 years** **(n=698)** | |
| Dementia | 2.27 (1.44–3.56) |
| Communication & speech problems | 1.92 (1.06–3.48) |
| Alzheimer’s disease | 1.69 (0.61–4.69) |
| Depression | 1.38 (1.01–1.87) |
| Falls | 1.37 (0.86–2.20) |
| Anxiety | 1.07 (0.78–1.46) |
| Attention deficit | 1.00 (0.35–2.88) |
| Sleep disturbances | 0.55 (0.38–0.79) |
| Stroke (ischemic) | 0.51 (0.24–1.07) |

Odds ratios are presented with 95% confidence intervals, only for prespecified comorbidities that were reported in ≥5 cases and ≥5 controls. Only the highest ten odds ratios are presented for each age group. CI: confidence interval; HD: Huntington’s disease; OCD: obsessive compulsive disorder; OR: odds ratio; PD: Parkinson’s disease.

Supplementary Table 4. Prespecified comorbidities stratified by age group: HD cases versus GP controls (analysis period only)

| **Comorbidity** | **OR (95% CI)** |
| --- | --- |
| **25–49 years** **(n=422)** | |
| Depression | 3.81 (2.42–6.00) |
| Falls | 2.33 (1.11–4.90) |
| Anxiety | 2.29 (1.48–3.54) |
| Sleep disturbances | 1.58 (0.91–2.74) |
| **≥50 years** **(n=698)** | |
| Communication & speech problems | 7.18 (2.77–18.63) |
| Falls | 6.31 (2.93–13.60) |
| Depression | 4.30 (2.97–6.22) |
| Anxiety | 2.67 (1.86–3.84) |
| Sleep disturbances | 1.57 (1.03–2.39) |

Odds ratios are presented with 95% confidence intervals, only for prespecified comorbidities that were reported in ≥5 cases and ≥5 controls. Only the highest ten odds ratios are presented for each age group. CI: confidence interval; GP: general population; HD: Huntington’s disease; OCD: obsessive compulsive disorder; OR: odds ratio.

Supplementary Table 5. Prespecified comorbidities stratified by age group: HD cases versus PD controls (analysis period and prior)

| **Comorbidity** | **OR (95% CI)** |
| --- | --- |
| **18–24 years** **(n=34)** | |
| Depression | 4.15 (0.86–19.92) |
| Communication & speech problems | 1.28 (0.32–5.13) |
| Sleep disturbances | 1.27 (0.33–4.93) |
| Anxiety | 0.61 (0.15–2.43) |
| Attention deficit | 0.60 (0.14–2.47) |
| **25–49 years** **(n=422)** | |
| Dementia | 3.99 (2.19–7.25) |
| Falls | 1.42 (0.90–2.24) |
| Anxiety | 1.21 (0.82–1.78) |
| Depression | 1.20 (0.81–1.78) |
| Attention deficit | 0.94 (0.47–1.88) |
| Irritability/aggression/violence | 0.90 (0.36–2.25) |
| Suicidality | 0.86 (0.45–1.61) |
| Sleep disturbances | 0.69 (0.47–1.02) |
| Communication & speech problems | 0.64 (0.42–0.97) |
| OCD | 0.53 (0.25–1.13) |
| **≥50 years** **(n=698)** | |
| Dementia | 2.60 (1.78–3.80) |
| Irritability/aggression/violence | 1.65 (0.67–4.03) |
| OCD | 1.51 (0.53–4.30) |
| Falls | 1.38 (0.98–1.92) |
| Stroke (haemorrhagic) | 1.35 (0.56–3.23) |
| Alzheimer’s disease | 1.30 (0.64–2.66) |
| Depression | 1.13 (0.83–1.53) |
| Suicidality | 1.00 (0.57–1.74) |
| Anxiety | 0.96 (0.71–1.29) |
| Attention deficit | 0.83 (0.41–1.67) |

Odds ratios are presented with 95% confidence intervals, only for prespecified comorbidities that were reported in ≥5 cases and ≥5 controls. Only the highest ten odds ratios are presented for each age group. CI: confidence interval; HD: Huntington’s disease; OCD: obsessive compulsive disorder; OR: odds ratio; PD: Parkinson’s disease.

Supplementary Table 6. Prespecified comorbidities stratified by age group: HD cases versus GP controls (analysis period and prior)

| **Comorbidity** | **OR (95% CI)** |
| --- | --- |
| **18–24 years** **(n=34)** | |
| Depression | 11.20 (2.20–56.93) |
| Falls | 2.89 (0.66–12.57) |
| Anxiety | 1.61 (0.41–6.24) |
| **25–49 years** **(n=422)** | |
| Depression | 3.09 (2.08–4.60) |
| Anxiety | 2.59 (1.75–3.84) |
| Attention deficit | 2.22 (0.94–5.27) |
| Suicidality | 1.90 (0.89–4.08) |
| Falls | 1.71 (1.07–2.75) |
| Communication & speech problems | 1.46 (0.92–2.31) |
| Sleep disturbances | 1.44 (0.96–2.16) |
| **≥50 years** **(n=698)** | |
| Suicidality | 4.79 (1.95–11.76) |
| Falls | 3.68 (2.44–5.54) |
| Depression | 2.94 (2.16–4.01) |
| Anxiety | 2.40 (1.76–3.28) |
| Sleep disturbances | 2.01 (1.46–2.76) |
| Stroke (ischemic) | 1.93 (0.99–3.75) |
| Communication & speech problems | 1.44 (1.04–2.01) |

Odds ratios are presented with 95% confidence intervals, only for prespecified comorbidities that were reported in ≥5 cases and ≥5 controls. Only the highest ten odds ratios are presented for each age group. CI: confidence interval; HD: Huntington’s disease; OCD: obsessive compulsive disorder; OR: odds ratio.

Supplementary Table 7. Overall comorbidities stratified by age group: HD cases versus PD controls (analysis period only)

| **Comorbidity (ICD code)** | **OR (95% CI)** |
| --- | --- |
| **2–17 years** **(n=20)** | |
| Encounter for general examination without complaint, suspected or reported diagnosis (Z00) | 0.67 (0.11–3.92) |
| **18–24 years** **(n=34)** | |
| Encounter for immunization (Z23) | 3.43 (0.83–14.21) |
| Encounter for general examination without complaint, suspected or reported diagnosis (Z00) | 0.62 (0.16–2.42) |
| **25–49 years** **(n=422)** | |
| Other and unspecified injuries of head (S09) | 4.18 (1.67–10.49) |
| Other degenerative diseases of nervous system, not elsewhere classified (G31) | 3.38 (1.22–9.40) |
| Other symptoms and signs involving the nervous and musculoskeletal systems (R29) | 3.08 (1.45–6.51) |
| Problems related to care provider dependency (Z74) | 2.72 (1.11–6.65) |
| Aphagia and dysphagia (R13) | 2.67 (1.46–4.88) |
| Other mental disorders due to known physiological condition (F06) | 2.51 (1.07–5.87) |
| Other lack of coordination (R27) | 2.08 (0.87–4.98) |
| Other disorders of external ear (H61) | 2.06 (0.76–5.60) |
| Unspecified mood [affective] disorder (F39) | 2.06 (0.76–5.60) |
| Symptoms and signs involving emotional state (R45) | 2.05 (1.02–4.12) |
| **≥50 years** **(n=698)** | |
| Dementia in other diseases classified elsewhere (F02) | 4.81 (2.51–9.19) |
| Speech disturbances, not elsewhere classified (R47) | 3.93 (1.68–9.19) |
| Symptoms and signs involving emotional state (R45) | 3.53 (1.82–6.87) |
| Unspecified mood [affective] disorder (F39) | 3.31 (1.20–9.13) |
| Pneumonitis due to solids and liquids (J69) | 2.66 (0.94–7.55) |
| Aphagia and dysphagia (R13) | 2.42 (1.58–3.72) |
| Other disorders of white blood cells (D72) | 2.18 (0.97–4.88) |
| Other sepsis (A41) | 2.17 (1.01–4.68) |
| Encounter for fitting and adjustment of other devices (Z46) | 2.05 (0.91–4.64) |
| Other disorders of conjunctiva (H11) | 2.03 (0.69–6.00) |

Odds ratios are presented with 95% confidence intervals, only for comorbidities that were reported in ≥5 cases and ≥5 controls. Only the highest ten odds ratios are presented for each age group. CI: confidence interval; HD: Huntington’s disease; ICD: International Classification of Diseases; OCD: obsessive compulsive disorder; OR: odds ratio; PD: Parkinson’s disease.

Supplementary Table 8. Overall comorbidities stratified by age group: HD cases versus GP controls (analysis period only)

| **Comorbidity (ICD code)** | **OR (95% CI)** |
| --- | --- |
| **2–17 years** **(n=20)** | |
| Encounter for general examination without complaint, suspected or reported diagnosis (Z00) | 1.00 (0.17–5.77) |
| **18–24 years** **(n=34)** | |
| Encounter for immunization (Z23) | 4.64 (1.06–20.39) |
| Encounter for general examination without complaint, suspected or reported diagnosis (Z00) | 2.13 (0.52–8.76) |
| **25–49 years** **(n=422)** | |
| Aphagia and dysphagia (R13) | 7.99 (3.31–19.30) |
| Other functional intestinal disorders (K59) | 6.83 (2.59–17.97) |
| Symptoms and signs involving emotional state (R45) | 5.54 (2.08–14.76) |
| Major depressive disorder, single episode (F32) | 4.25 (2.46–7.34) |
| Other symptoms and signs involving cognitive functions and awareness (R41) | 3.73 (1.79–7.78) |
| Major depressive disorder, recurrent (F33) | 3.35 (1.73–6.49) |
| Other and unspecified injuries of head (S09) | 3.10 (1.36–7.11) |
| Persons encountering health services in other circumstances (Z76) | 2.99 (1.16–7.75) |
| Schizophrenia (F20) | 2.93 (1.04–8.28) |
| Dermatophytosis (B35) | 2.58 (1.20–5.54) |
| **≥50 years** **(n=698)** | |
| Aphagia and dysphagia (R13) | 13.60 (6.17–29.98) |
| Symptoms and signs involving emotional state (R45) | 8.66 (3.37–22.24) |
| Other symptoms and signs involving cognitive functions and awareness (R41) | 8.32 (4.07–17.03) |
| Abnormalities of gait and mobility (R26) | 8.08 (4.31–15.15) |
| Personal risk factors, not elsewhere classified (Z91) | 6.71 (2.58–17.46) |
| Other extrapyramidal and movement disorders (G25) | 5.99 (2.77–12.94) |
| Dermatophytosis (B35) | 5.80 (2.89–11.63) |
| Volume depletion (E86) | 4.85 (1.82–12.92) |
| Other disorders of fluid, electrolyte and acid-base balance (E87) | 4.43 (2.25–8.74) |
| Other and unspecified injuries of head (S09) | 4.26 (1.84–9.89) |

Odds ratios are presented with 95% confidence intervals, only for comorbidities that were reported in ≥5 cases and ≥5 controls. Only the highest ten odds ratios are presented for each age group. CI: confidence interval; GP: general population; HD: Huntington’s disease; ICD: International Classification of Diseases; OCD: obsessive compulsive disorder; OR: odds ratio.

Supplementary Table 9. Overall comorbidities stratified by age group: HD cases versus PD controls (analysis period and prior)

| **Comorbidity (ICD code)** | **OR (95% CI)** |
| --- | --- |
| **2–17 years** **(n=20)** | |
| Symptoms involving respiratory system and other chest symptoms (786) | 2.33 (0.37–14.61) |
| General symptoms (780) | 2.25 (0.17–29.77) |
| Symptoms involving nervous and musculoskeletal systems (781) | 1.56 (0.24–9.91) |
| Symptoms involving digestive system (787) | 1.56 (0.24–9.91) |
| Encounter for immunization (Z23) | 1.50 (0.26–8.82) |
| Other disorders of soft tissues (729) | 1.50 (0.26–8.82) |
| Acute pharyngitis (462) | 1.00 (0.17–5.98) |
| Acute upper respiratory infections of multiple or unspecified sites (465) | 1.00 (0.15–6.77) |
| Encounter for general examination without complaint, suspected or reported diagnosis (Z00) | 0.38 (0.05–2.77) |
| Health supervision of infant or child (V20) | 0.26 (0.02–3.06) |
| **18–24 years** **(n=34)** | |
| Other extrapyramidal disease and abnormal movement disorders (333) | 22.86 (2.43–214.56) |
| Depressive disorder, not elsewhere classified (311) | 2.70 (0.66–11.09) |
| Encounter for immunization (Z23) | 2.13 (0.52–8.76) |
| Other symptoms involving abdomen and pelvis (789) | 2.06 (0.52–8.17) |
| Acute upper respiratory infections of multiple or unspecified sites (465) | 2.04 (0.52–8.00) |
| Symptoms concerning nutrition metabolism and development (783) | 1.68 (0.41–6.96) |
| Symptoms involving nervous and musculoskeletal systems (781) | 1.61 (0.41–6.24) |
| Viral and chlamydial infection in conditions classified elsewhere and of unspecified site (79) | 1.31 (0.31–5.53) |
| General medical examination (V70) | 1.28 (0.32–5.13) |
| Allergic rhinitis (477) | 1.00 (0.24–4.08) |
| Bacterial infection in conditions classified elsewhere and of unspecified site (41) | 1.00 (0.23–4.37) |
| Encounter for general examination without complaint, suspected or reported diagnosis (Z00) | 1.00 (0.26–3.92) |
| Encounter for other and unspecified procedures and aftercare (V58) | 1.00 (0.26–3.85) |
| Other and unspecified disorders of joint (719) | 1.00 (0.26–3.92) |
| Specific delays in development (315) | 1.00 (0.24–4.08) |
| **25–49 years** **(n=422)** | |
| Dementia in other diseases classified elsewhere (F02) | 9.01 (3.49–23.50) |
| Other extrapyramidal disease and abnormal movement disorders (333) | 6.38 (4.16–9.79) |
| Open wound of head (S01) | 3.88 (1.73–8.73) |
| Other mental disorders due to known physiological condition (F06) | 3.19 (1.60–6.34) |
| Other degenerative diseases of nervous system, not elsewhere classified (G31) | 2.95 (1.28–6.79) |
| Family history of certain chronic disabling diseases (V17) | 2.54 (1.25–5.14) |
| Alcohol-related disorders (F10) | 2.43 (0.91–6.44) |
| Unspecified fall (W19) | 2.14 (1.07–4.29) |
| Bronchitis, not specified as acute or chronic (J40) | 2.07 (0.82–5.24) |
| Problems related to care provider dependency (Z74) | 2.03 (0.98–4.19) |
| **≥50 years** **(n=698)** | |
| Dementias (290) | 4.85 (1.82–12.92) |
| Other extrapyramidal disease and abnormal movement disorders (333) | 4.85 (3.50–6.71) |
| Dementia in other diseases classified elsewhere (F02) | 4.20 (2.49–7.08) |
| Conduct disorders (F91) | 4.18 (1.55–11.27) |
| Protein-calorie malnutrition of moderate and mild degree (E44) | 3.96 (1.46–10.73) |
| Complications of artificial openings of the digestive system (K94) | 3.74 (1.37–10.19) |
| Spinocerebellar disease (334) | 3.29 (1.30–8.34) |
| Specific personality disorders (F60) | 2.75 (1.06–7.10) |
| Persistent mental disorders due to conditions classified elsewhere (294) | 2.72 (1.72–4.29) |
| Family history of certain disabilities and chronic diseases (leading to disablement) (Z82) | 2.67 (1.22–5.85) |
| Encounter for attention to artificial openings (Z43) | 2.50 (1.02–6.11) |

Odds ratios are presented with 95% confidence intervals, only for comorbidities that were reported in ≥5 cases and ≥5 controls. Only the highest ten odds ratios are presented for each age group. CI: confidence interval; GP: general population; HD: Huntington’s disease; ICD: International Classification of Diseases; OCD: obsessive compulsive disorder; OR: odds ratio; PD: Parkinson’s disease.

Supplementary Table 10. Overall comorbidities stratified by age group: HD cases versus GP controls (analysis period and prior)

| **Comorbidity (ICD code)** | **OR (95% CI)** |
| --- | --- |
| **2–17 years (n=20)** | |
| Encounter for immunization (Z23) | 1.50 (0.26–8.82) |
| Acute pharyngitis (462) | 0.64 (0.10–4.10) |
| Acute upper respiratory infections of multiple or unspecified sites (465) | 0.58 (0.07–4.56) |
| Health supervision of infant or child (V20) | 0.26 (0.02–3.06) |
| Encounter for general examination without complaint, suspected or reported diagnosis (Z00) | 0.17 (0.01–1.88) |
| **18–24 years (n=34)** | |
| Depressive disorder, not elsewhere classified (311) | 5.76 (1.32–25.19) |
| General symptoms (780) | 4.64 (1.06–20.39) |
| Encounter for immunization (Z23) | 4.40 (1.04–18.60) |
| Other symptoms involving abdomen and pelvis (789) | 2.62 (0.65–10.48) |
| Special investigations and examinations (V72) | 2.13 (0.52–8.76) |
| Encounter for general examination without complaint, suspected or reported diagnosis (Z00) | 2.04 (0.52–8.00) |
| Encounter for other and unspecified procedures and aftercare (V58) | 1.63 (0.41–6.46) |
| Other disorders of soft tissues (729) | 1.28 (0.32–5.13) |
| Other and unspecified disorders of joint (719) | 1.27 (0.33–4.93) |
| Acute upper respiratory infections of multiple or unspecified sites (465) | 1.00 (0.26–3.92) |
| General medical examination (V70) | 1.00 (0.26–3.92) |
| **25–49 years (n=422)** | |
| Other extrapyramidal disease and abnormal movement disorders (333) | 136.21 (53.05–349.72) |
| Abnormalities of gait and mobility (R26) | 11.05 (5.14–23.74) |
| Family history of certain chronic disabling diseases (V17) | 6.30 (2.38–16.67) |
| Aphagia and dysphagia (R13) | 6.25 (3.16–12.36) |
| Symptoms involving nervous and musculoskeletal systems (781) | 5.80 (3.23–10.41) |
| Other symptoms and signs involving cognitive functions and awareness (R41) | 5.11 (2.69–9.71) |
| Symptoms and signs involving emotional state (R45) | 4.54 (2.04–10.10) |
| Personal risk factors, not elsewhere classified (Z91) | 4.28 (1.82–10.05) |
| Other functional intestinal disorders (K59) | 3.79 (1.97–7.29) |
| Major depressive disorder, single episode (F32) | 3.62 (2.30–5.67) |
| **≥50 years (n=698)** | |
| Other extrapyramidal disease and abnormal movement disorders (333) | 68.09 (38.44–120.62) |
| Aphagia and dysphagia (R13) | 14.91 (7.85–28.29) |
| Other symptoms and signs involving the nervous and musculoskeletal systems (R29) | 13.43 (5.31–33.95) |
| Abnormalities of gait and mobility (R26) | 12.38 (6.94–22.08) |
| Other degenerative diseases of nervous system, not elsewhere classified (G31) | 12.05 (4.75–30.55) |
| Other symptoms and signs involving cognitive functions and awareness (R41) | 11.27 (6.19–20.53) |
| Other nonorganic psychoses (298) | 10.18 (3.99–25.99) |
| Symptoms and signs involving emotional state (R45) | 9.34 (4.19–20.80) |
| Superficial injury of head (S00) | 8.41 (3.27–21.63) |
| Other and unspecified injuries of head (S09) | 8.16 (3.99–16.71) |

Odds ratios are presented with 95% confidence intervals, only for comorbidities that were reported in ≥5 cases and ≥5 controls. Only the highest ten odds ratios are presented for each age group. CI: confidence interval; GP: general population; HD: Huntington’s disease; ICD: International Classification of Diseases; OCD: obsessive compulsive disorder; OR: odds ratio.

Supplementary Table 11. Odds ratios for prespecified comorbidities not listed in Figure 3

| **Comorbidity** | **OR (95% CI)** |
| --- | --- |
| **HD vs PD (analysis period only)** | |
| Sleep disturbances | 0.67 (0.51–0.89) |
| Stroke (ischemic) | 0.37 (0.18–0.74) |
| **HD vs PD (analysis period and prior)** | |
| OCD | 0.78 (0.44–1.38) |
| Myocardial infarction | 0.77 (0.33–1.76) |
| Sleep disturbances | 0.69 (0.55–0.87) |
| Communication & speech problems | 0.67 (0.52–0.85) |
| Stroke (ischemic) | 0.44 (0.28–0.69) |
| **HD vs GP (analysis period and prior)** | |
| Myocardial infarction | 1.44 (0.54–3.80) |

CI: confidence interval; OR: odds ratio.

Supplementary Table 12. Odds ratios for overall comorbidities not listed in Figure 4

| **Comorbidity (ICD code)** | **OR (95% CI)** |
| --- | --- |
| **HD vs PD (analysis period only)** | |
| Mental disorder, not otherwise specified (F99) | 2.29 (0.99–5.31) |
| Pneumonitis due to solids and liquids (J69) | 2.27 (1.02–5.02) |
| Injury of unspecified body region (T14) | 2.05 (1.07–3.94) |
| Encounter for attention to artificial openings (Z43) | 2.03 (0.86–4.78) |
| Delirium due to known physiological condition (F05) | 2.02 (0.69–5.94) |
| Leiomyoma of uterus (D25) | 2.02 (0.69–5.94) |
| Unspecified dementia (F03) | 1.88 (1.15–3.08) |
| Unspecified mental disorder due to known physiological condition (F09) | 1.88 (0.74–4.74) |
| Other mental disorders due to known physiological condition (F06) | 1.83 (1.12–3.01) |
| Other disorders of external ear (H61) | 1.82 (1.01–3.29) |
| **HD vs GP (analysis period only)** | |
| Other functional intestinal disorders (K59) | 4.28 (2.58–7.10) |
| Dermatophytosis (B35) | 4.15 (2.50–6.90) |
| Major depressive disorder, single episode (F32) | 4.15 (3.00–5.73) |
| Volume depletion (E86) | 4.07 (1.94–8.55) |
| Other disorders of brain (G93) | 4.04 (1.84–8.86) |
| Unspecified urinary incontinence (R32) | 3.95 (1.60–9.77) |
| Hypotension (I95) | 3.95 (1.60–9.77) |
| Retention of urine (R33) | 3.77 (1.52–9.37) |
| Other and unspecified injuries of head (S09) | 3.71 (2.06–6.66) |
| Mental disorder, not otherwise specified (F99) | 3.68 (1.36–9.98) |
| **HD vs PD (analysis period and prior)** | |
| Other mental disorders due to known physiological condition (F06) | 2.28 (1.51–3.43) |
| Unspecified dementia (F03) | 2.27 (1.47–3.52) |
| Fracture of skull and facial bones (S02) | 2.19 (0.83–5.81) |
| Dyslexia and other symbolic dysfunctions, not elsewhere classified (R48) | 2.16 (0.92–5.04) |
| Family history of certain chronic disabling diseases (V17) | 2.14 (1.40–3.26) |
| Aphagia and dysphagia (R13) | 2.06 (1.56–2.72) |
| Vascular dementia (F01) | 2.02 (0.75–5.42) |
| Speech disturbances, not elsewhere classified (R47) | 1.90 (1.26–2.87) |
| Conduct disorders (F91) | 1.88 (1.10–3.20) |
| Gingivitis and periodontal diseases (K05) | 1.85 (0.68–5.03) |
| **HD vs GP (analysis period and prior)** | |
| Superficial injury of head (S00) | 7.31 (3.72–14.36) |
| Symptoms and signs involving emotional state (R45) | 7.09 (4.05–12.39) |
| Other epidermal thickening (L85) | 6.49 (2.51–16.81) |
| Symptoms involving nervous and musculoskeletal systems (781) | 6.30 (4.49–8.85) |
| Artificial opening status (Z93) | 6.05 (2.33–15.74) |
| Disturbance of conduct not elsewhere classified (312) | 6.01 (2.81–12.86) |
| Somnolence, stupor and coma (R40) | 5.85 (2.85–12.02) |
| Mental disorder, not otherwise specified (F99) | 5.58 (2.32–13.46) |
| Other nonorganic psychoses (298) | 5.54 (3.08–9.96) |
| Other specified and unspecified injuries of neck (S19) | 5.40 (2.24–13.04) |

CI: confidence interval; OR: odds ratio.
